# Meningioma: A Review of Clinicopathological and Molecular Aspects

**DOI:** 10.3389/fonc.2020.579599

**Published:** 2020-10-23

**Authors:** Kristin Huntoon, Angus Martin Shaw Toland, Sonika Dahiya

**Affiliations:** ^1^ Department of Neurological Surgery, The Ohio State University Wexner Medical Center, Columbus, OH, United States; ^2^ Department of Pathology, Stanford University, Stanford, CA, United States; ^3^ Department of Pathology and Immunology, Washington University School of Medicine, St. Louis, MO, United States

**Keywords:** meningioma, targeted treatment, molecular diagnosis, immunotherapy, neurosurgery, clinical trials, pathology, radiation therapy

## Abstract

Meningiomas are the most the common primary brain tumors in adults, representing approximately a third of all intracranial neoplasms. They classically are found to be more common in females, with the exception of higher grades that have a predilection for males, and patients of older age. Meningiomas can also be seen as a spectrum of inherited syndromes such as neurofibromatosis 2 as well as ionizing radiation. In general, the 5-year survival for a WHO grade I meningioma exceeds 80%; however, survival is greatly reduced in anaplastic meningiomas. The standard of care for meningiomas in a surgically-accessible location is gross total resection. Radiation therapy is generally saved for atypical, anaplastic, recurrent, and surgically inaccessible benign meningiomas with a total dose of ~60 Gy. However, the method of radiation, regimen and timing is still evolving and is an area of active research with ongoing clinical trials. While there are currently no good adjuvant chemotherapeutic agents available, recent advances in the genomic and epigenomic landscape of meningiomas are being explored for potential targeted therapy.

## Introduction

### Epidemiology

Meningiomas arise from arachnoid cap cells in the brain, and represent 37.6% of all primary brain tumors in adults, making them the most common type of intracranial tumor with an incidence of 8.83 per 100,000 in the most recent Central Brain Tumor Registry of the United States (1, 2). Conversely, they are equally rare in children and adolescents of both sexes (0.4%–4.6%) (3). The median age of diagnosis of meningioma is 65 years, likely due to the increasing incidence of meningiomas with age (2). Additionally, in the adult population, there is a marked female bias with a female:male ratio of 3:1 and increasing to 9:1 for spinal lesions (3). The rate of diagnosis of meningiomas has increased due to better imaging facilities and ageing populations with one survey showing a 3.9-fold increase in diagnosis of meningioma since 1943 (4). The calculated lifetime risk of developing meningioma without any associated factors is approximately 1% (3).

### Clinical History

As with many of the lesions of the CNS, the symptoms correspond to the location of the mass. Meningiomas are slow growing and often not infiltrative in nature thus the symptoms tend to be insidious in onset. Common presentations include headaches secondary to increased intracranial pressure, focal neurological (cranial nerve) deficits, and seizures caused by mass effect and/or direct involvement by the tumor (5). A rare clinical syndrome, Foster Kennedy syndrome coined in 1911 by Dr. Robert Foster Kennedy, is characterized by ipsilateral optic atrophy, papilledema in contralateral eye, central scotoma in ipsilateral eye, and anosmia, secondary to a large olfactory meningioma (6). Large frontal meningiomas may also present with personality changes or altered mental status which can lead to a misdiagnosis of dementia or severe depression (5).

### Natural History

Understanding the natural history of meningiomas is imperative for clinicians with a growing amount of incidental meningiomas now detected secondary to advanced imaging studies. As mentioned previously, meningiomas are generally slow growing lesions with a linear growth rate of 2–4 mm/year for asymptomatic meningiomas (7). In a retrospective study in which incidental meningiomas were followed by imaging, approximately a third of the tumors did not grow at all. However, of those that grew, nearly 25% grew exponentially, further underscoring the importance of surveillance imaging in untreated patients (8). The natural course of symptomatic larger lesions is deemed anecdotally to be a more aggressive growth pattern, but these lesions are rarely left untreated, and therefore, their true natural history remains ill-defined (9).

The estimated 10-year survival (overall 61.7%) for malignant meningiomas is very much dependent on age; 10-year relative survival is estimated to be around 76.8% for 20–44 year olds, while it is only 39.5% for patients age 75 years and older (2). Malignant meningioma of the spine has a higher 10-year relative survival of 73.4% when compared to the survival rate of 55.7% for intracranial tumors. Recurrence is a function of surgical resection (and/or radiation typically as adjuvant therapy in a subset), location and the histological grade of the meningioma (2), although location and surgical resection are somewhat interlinked. In terms of recurrence differences with grade, the five-year progression free survival (PFS) for a WHO grade I tumors is ~90% after gross total resection (GTR), Grade II are ~ 60%–90%, whereas grade III PFS after GTR is 28% (10, 11). These recurrences translate into meningioma-specific mortality in these patients, with 10-year overall survival rates of 53% for grade II patients and 0% for grade III patients, despite aggressive therapeutic efforts (12).

### Etiology

#### Syndromes

Interestingly, in children and adolescents, meningiomas show a tendency for more aggressive subtypes. This may be secondary to their occurrence in several associated hereditary syndromes, such as Neurofibromatosis type 2 (NF-2) most commonly, but also less common causes such as Gorlin syndrome and Cowden syndrome (13).

Loss of heterozygosity and inactivating mutations in the *NF2* gene are seen in up to 60% of sporadic cases (14, 15). Germline mutations in the same gene lead to neurofibromatosis 2 (NF2), an autosomal disorder characterized by the occurrence of schwannomas, mengingiomas, and gliomas. The mutation often presents as a cytogenetically visible deletion of the long arm of chromosome 22 at q12, leading to decreased functional levels of the tumor suppressor gene, Merlin. More than half of patients with NF2 will demonstrate at least one meningioma in their lifetime, with initial diagnosis at the mean age of 30 (16, 17). The associated risk of the meningioma corresponds to the type of mutation seen. For example, a truncating mutation by frameshift tends to cause a greater tumor burden with early initial onset of meningioma. Most NF2 related meningiomas present as a fibrous or transitional phenotype, which are the most common histopathological subtypes of meningioma and are generally more aggressive than sporadic tumors (18, 19).

Gorlin syndrome or nevoid basal cell carcinoma syndrome is an uncommon autosomal dominant disease with an estimated prevalence varying from 1/57,000 to 1/256,000, and affecting males and females equally (20). Inactivation of the *PTCH1* gene located on chromosome 9q22.3-q31 is the hallmark of Gorlin syndrome. A second hit mutation of p53 often results in the formation of multiple BCC. *PTCH1* gene mutations lead to a hypersensitivity to radiation-induced tumorigenesis (21). Another missense mutation of the downstream factor, *SUFU* can be found rarely in families with hereditary multiple meningiomas (22). A natural history study from NIH speculated that patients affected by Gorlin syndrome have a 5% incidence of having a CT with radiological features suggestive of meningioma (23).

Cowden syndrome is an autosomal-dominant syndrome that predisposes the patient to developing benign and malignant cancers of a variety of organ systems, including breast, thyroid, uterus, and CNS. It is characterized by multiple hamartomas of ectodermal, mesodermal, and endodermal origin (24). Cytogenetically, it is associated with deletions on the chromosome 10 (PTEN) gene on 10q23.31 (25, 26). It shows a strong female dominance with an overall prevalence of one in 200,000 (27). The incidence of meningioma in patients with CS was 8.25% in a systematic meta-analysis (28).

Several hereditary conditions are associated with germline mutation of the SMARCB1 gene on 22q11.23, including schwannomatosis, rhabdoid tumor predisposition syndrome [atypical teratoid/rhabdoid tumor (AT/RT)], and Coffin-Siris syndrome.

Germline mutation of the *SMARCB1* gene on 22q11.23 causes several hereditary conditions, such as rhabdoid tumor predisposition syndrome (AT/RT) (29), schwannomatosis (30), and Coffin-Siris syndrome (31). Schwannomatosis is associated with a nontruncating mutation at the beginning of end of the SMARCB1 gene, presenting as a bening tumor disposition syndrome (32); 5% of patients with this syndrome will develop a meningioma. SMARCB1 is very closely associated to NF2 on chromosome 22, and co-mutation of both genes has been seen with tumorigenesis of meningiomas (33). Germline mutations of *SMARCE1* gene on 17q21.2, with nearly all mutations being truncating characterized by loss of function mutations, was identified in families with multiple spinal meningiomas, and later alterations in *SMARCE1* were also found in individuals with intracranial and spinal clear cell meningiomas (34, 35).

BRCA1-associated protein 1 (BAP1) tumor predisposition syndrome (BAP1-TPDS) is associated with a germline mutation of the *BAP1* gene on 3p21.1. These individuals are vulnerable to a variety of neoplasms, including uveal and cutaneous melanomas, pleural and peritoneal mesotheliomas, renal cell carcinoma, and mesothelioma (36). Those affected develop meningiomas by the time they reach 50 years of age (37). Meningiomas in BAP1-TPDS tend to demonstrate rhabdoid morphology and show aggressive clinical behavior (36). BAP1 encodes a ubiquitin carboxyl-terminal hydrolase 1, which is involved in the regulation of chromatin modification as a part of the polycomb repressive complex (PRC), and response to DNA damage by interacting with a tumor suppressor, BRCA1 (36).

Other familial syndromes associated with meningiomas include Rubinstein-Taybi syndrome, Li-Fraumeni syndrome, Gardner syndrome, multiple endocrine neoplasia type 1, and Werner syndrome (**Table 1**).

**Table 1:** Familial syndromes associated with meningiomas.

Familial syndrome	Gene	Chromosome locus
Neurofibromatosis type 2	*NF2*	22q12
Familial schwannomatosis	*SMARCB1*	22q11.23
Multiple spinal meningiomas	*SMARCE1*	17q21.2
BAP1 tumor predisposition syndrome	*BAP1*	3p21.1
Gorlin syndrome (nevoid basal cell carcinoma syndrome)	*PTCH1*	9q22.3
	*SUFU*	10q24.32
Familial multiple meningiomas	*SUFU*	10q24.32
Rubinstein-Taybi syndrome	*CREBBP*	16p13.3
Cowden disease	*PTEN*	10q23.31
Li-Fraumeni syndrome	*TP53/CHEK2*	17p13.1/22q12.1
Gardner syndrome	*APC*	5q21-22
Multiple endocrine neoplasia type 1	*MEN*	11q13
Werner syndrome	*LMNA*	1q21.1

Specific gene and chromosome locus are illustrated in the table.

#### Radiation

A primary modifiable risk factor for the development of meningioma is exposure to ionizing radiation, resulting in a six- to 10-fold increase in risk (38). For example, individuals who underwent low dose radiation (1-6 Gy) for the treatment of tinea capitis of the scalp were found to have a 2.3% lifetime risk over 35 years of developing one or more meningiomas (39). Likewise, a large study conducted by the USA Childhood Cancer Survivor Study (CCSS) reported on the incidence of secondary malignancy estimated to be 3.1% for meningioma alone, in which radiation exposure was identified as an independent risk factor, with a relative risk of 2.7 (40). In a meta-analysis, the mean intervals between primary cancer diagnosis (90% acute lymphoblastic leukemia (ALL) or brain tumor) and subsequent meningioma diagnosis were 10.7 to 23.1 years (41). Of note, these radiation induced meningioma have been found to have more atypical features with a high proliferation index resulting in a higher grade meningioma as well as being multifocal in nature (42). However, a review of survivorship data found that 5-year survival rates were similar to those with primary meningiomas (41, 42). Among the survivors of the atomic bomb in Hiroshima the incidence of meningiomas found on imaging in 5-year intervals since 1975 were 5.3, 7.3, 10.1, and 14.9 cases per 10 (5) population, respectively (43). Data from the Hiroshima Tumor Registry also showed that the incidence of meningioma was relative to the distance of radiation source, showing that individuals exposed within 1 km had three times higher risk than among those exposed 2 km away (43).

#### Hormone Receptors

There has long been an association with hormone receptors expressed on meningiomas and their increased frequency among female patients, although the data has been highly variable. In a large scale study of ~500 meningiomas, 88% were progesterone receptor positive, 40% were positive for estrogen and 39% for androgen receptors. Estrogen and androgen receptors were significantly more common on lower grade (Grade I) meningiomas compared to higher grade lesions. In addition, estrogen-positive tumor samples showed a higher proliferation index than those that were estrogen-negative (44). However, a population-based, matched case-control study showed no significant associated between the risk of meningioma and the use of exogenous hormones (such as oral contraceptive use or hormone replacement therapy) (45).

### Location

Meningiomas are thought to arise from meningothelial cells (arachnoid “cap” cells) and occur more frequently in areas where cap cells are most numerous. Cap cells are especially concentrated in the arachnoid granulations and are a common site of origin for meningiomas, especially along the dural venous sinuses where villi of arachnoid granulations are clustered. Additional sites of origin include the arachnoid associated with cranial nerves as they exit the cranial vault and even the choroid plexus (since the arachnoid participates in its formation, i.e., tela choroidea). Lesions in spinal locations constitute approximately 12% of all meningiomas. Of intracranial and juxtacranial meningiomas, the most to least common locations for occurrence of meningioma are: convexity (lateral hemisphere) (20%–34%); parasagittal (medial area of hemispheres) (18%–22%) (includes falcine meningiomas [5%], which account for lesions adjacent/involving the superior sagittal sinus or in some cases extending to both sides of sinus); sphenoid and middle cranial fossa (17%–25%); frontobasal (10%); posterior fossa (9%–15%), including the tentorium cerebelli (2%–4%), cerebellar convexity (5%), cerebellopontine angle (2%–4%), and clivus (< 1%); intraventricular (2%–5%) and orbital (<1%–2%) (**Table 2**) (46, 47). Recognizing potential atypical locations of these neoplasms is critical to ensure both proper diagnosis and treatment.

**Table 2:** Frequency of meningioma depending upon location.

Location	Frequency
Convexity	20–34%
Parasagittal	18–22%
Falcine	5%
Sphenoid and middle cranial fossa	17–25%
Frontobasal	10%
Posterior fossa	9–15%
Tentorium cerebelli	2–4%
Cerebellar convexity	5%
Cerebellopontine angle	2–4%
Clivus	<1%
Intraventricular	2–5%
Orbital	<1–2%
Ectopic	<1%

### Imaging Characteristics

**Figure 1:**
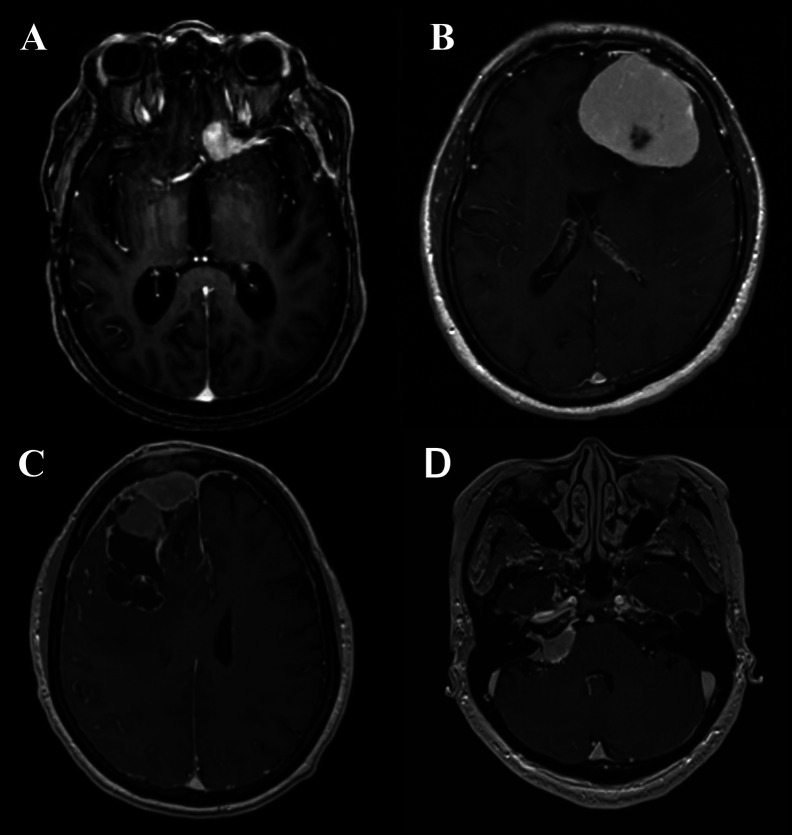
**(A)** Axial T1-post contrast MRI demonstrating an anterior clinoid meningioma with a characteristic dural tail. **(B)** Axial T1-post contrast MRI demonstrating a convexity meningioma with dural tails. **(C)** Axial T1-post contrast MRI demonstrating a meningioma with irregular edges abutting the superior sagittal sinus. **(D)** Axial T1-post contrast MRI demonstrating cerebellopontine angle meningioma.

The standard modality of radiological diagnosis of meningiomas is magnetic resonance imaging (MRI). However, in the cases in which a patient cannot undergo an MRI (e.g., pacemaker or other MRI incompatible device), a contrast-enhanced computed tomography (CT) may be utilized. Of meningiomas harbor regions of intralesional calcifications which can be observed, as well as bony changes, including hyperostosis resulting in a “beaten brass” appearance of the remodeled skull, this is particularly true of lesions along the sphenoid wings and convexity which can be more avidly seen on CT imaging. On MRI meningiomas may have the hallmark dural tail, and overall the lesion should have homogeneous enhancement and be well-circumscribed (**Figure 1**). Benign lesions additionally are isodense to surrounding gray matter on noncontract sequences. Nearly all meningiomas are extraaxial in nature and some may have CSF cleft adjacent to the meningioma. The majority of patients with meningiomas present with a solitary tumor, multiple meningiomas may be seen, particularly in NF2, however, multiple extra axial lesions could also be a result of metastatic disease (48).

Although the dural tail mentioned before is a hallmark of a meningioma—it is not pathognomonic and may also be observed with metastases or solitary fibrous tumor/hemangiopericytoma, but is frequently useful in distinguishing meningioma from other lesions (such as schwannoma) where it is absent (48). Infrequently peritumoral edema on T2 or FLAIR imaging may also be noted, in more aggressive meningiomas and in association with secretory and angiomatous histological phenotypes (12). Areas of central necrosis (hypointense T1, nonenhancing, cystic appearing) are not specific for malignant or higher grade meningiomas this finding can be seen on imaging in lower grade lesions as well (48). In fact, necrosis can commonly be seen after intravascular embolization of the meningiomas, which can be utilized and warranted in meningiomas that appear hypervascular pre-operatively to decrease blood loss. Lesions of the skull base may abut or encase the carotid or basilar arteries and their respective branches, and often an MR angiogram will be obtain to visualize these structures prior to any treatment. Likewise, MR Venograms are thus used for parafalcine meningiomas that are near or involving the superior sagittal sinus to determine if the lesion has direct invasion of the sinus, is causing sinus compression secondarily to mass effect, or has caused thrombosis of the sinus. Despite advancements in MR aiding in the diagnosing of meningiomas, it is not yet predictive of pathological grade or other measure of the aggressive nature of the lesion. Some have shown that there is an inverse correlation between the ADC and Ki-67 proliferation index values in meningiomas, and thus associate the ADC values of the low-grade and high-grade meningiomas (49). PET imaging technology is serving to circumvent the some of the issues with MRI to discern early recurrence versus treatment-related radiographic changes with utilization of a 68- Gallium-labeled somatostatin-receptor analogue (68-Ga-DOTATE) (50). Another PET imaging advancement has been the utilization of tryptophan metabolism *via* α-[(11)C]-methyl-L-tryptophan PET (AMT-PET), in which early studies has been shown that it may be able to delineate tumor grade among meningiomas and other primary brain tumors (51). However, in AMT-PET the (11) C labeled for visualization has a half-life of only 20 minutes (52). As with other primary brain tumors, MR spectroscopy (MRS) studies have features of increased choline peak combined with decreased N-acetyl aspartate and creatinine peaks in comparison with normal brain (53). A distinct alanine peak is a hallmark of meningiomas with variable sensitivity (54). The presence of alanine in meningiomas may be due to partial oxidation of glutamine (55) or conversion from an increased pool of pyruvate secondary to inhibitions of the enzyme pyruvate kinase by l-alanine (56). As with the other technologies listed the ability of MRS to determine to tumor grade is not well established; however, it has been shown that an elevated lactate more often seen in atypical meningioma. Likewise, the absolute concentrations of total alanine and creatine have been shown to be decreased in high-grade when compared with low-grade meningiomas, as was the ratio of glycine to alanine (57).

### Pathology

Antoine Louis in 1774, a French surgeon, described a tumor-like meningioma and called it “fungus durae matris”. However, it was Harvey Cushing an American neurosurgeon that was the first to use the term “meningioma” in 1922 (58, 59). Dr. Kepes’s work on the tumor’s biology, pathology and differential diagnoses has further helped advance this field (60). The histologic feature of a meningioma that is pathognomonic is “whorl” formation by meningothelial cells, which can mineralize to harbor “psammoma bodies” (concentric dystrophic calcifications). Additionally intranuclear cytoplasmic pseudoinclusions, which are cytoplasmic invaginations in the nuclei, nuclear clearing and nuclear grooves are often observed. Nonetheless, these features can be absent or often unassuming in a subset of meningiomas. Immunohistochemistry may be utilized for confirmation in such examples, with the most widely marker being epithelial membrane antigen (EMA). More recent studies have clearly shown that somatostatin receptor 2A (SST2A) is a superior immunostain target due to its higher sensitivity (61).

**Figure 2:**
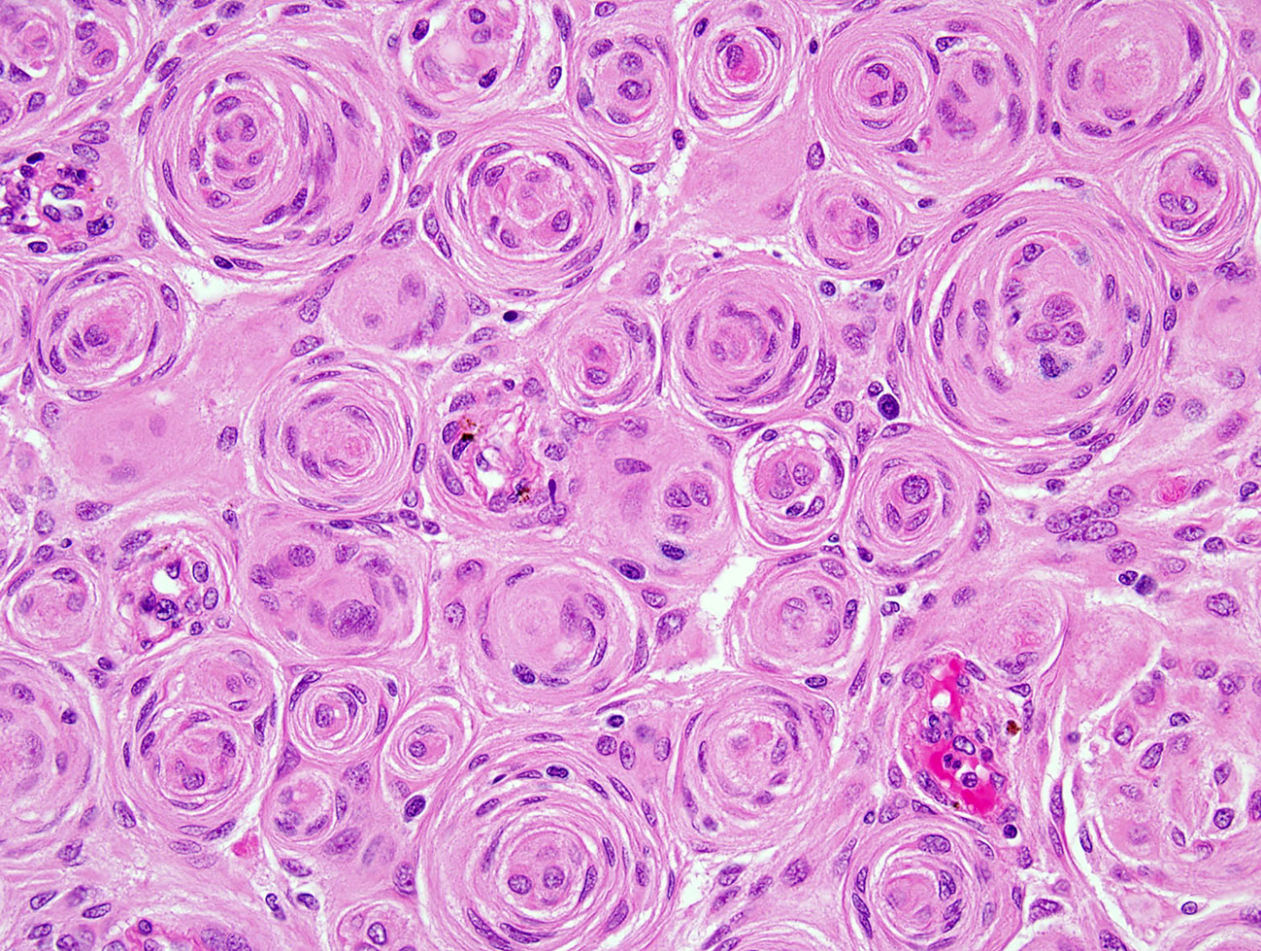
H&E of meningothelial meningioma with prominent whorled architecture (400×; H&E, hematoxylin and eosin stain).

Meningiomas are heterogeneous in their histopathologic features. Currently, 15 variants exist that are classified into three histologic grades. The WHO grade I (benign) includes nine variants, and the most frequent are meningothelial (**Figure 2**), fibrous, and transitional variants. Psammomatous, angiomatous, microcystic, secretory, lymphoplasmacyte-rich, and metaplastic variants are also included in grade I. Atypical, chordoid, and clear cell variants are included in grade II, whereas anaplastic, papillary, and rhabdoid variants are included in grade III (**Table 3**).

**Table 3:** WHO Grade with their associated histopathological subtypes.

Grade	Histopathologic features
**WHO Grade I**	
	Meningothelial
	Fibrous (fibroblastic)
	Microcystic
	Transitional
	Psammomatous
	Angiomatous (includes hemangioblastic, angioblastic)
	Secretory subtypes
	Metaplastic
	Lymphoplasmacyte rich
**WHO Grade II**	
	Clear cell
	Choroid
	Atypical
**WHO Grade III**	
	Rhabdoid
	Papillary
	Anaplastic

Meningiomas are classified as grade II “atypical” tumors if the lesion contains 4 or more mitoses per 10 consecutive high-power fields (using a 40× objective) or brain invasion, latter defined as meningioma infiltration into the underlying brain parenchyma without an intervening layer of connective tissue (62). In prior WHO classifications, invasion was considered a staging feature rather than a grading feature; however, it is recognized in the new grading that the presence of brain invasion in a WHO grade I meningioma confers recurrence and mortality rates similar to those of a WHO grade II meningioma (63). If neither feature is present, at least three of the following five histologic criteria must be evident to arrive at a grade II diagnosis: spontaneous intratumoral necrosis; patternless pattern or sheeted architecture; prominent nucleoli; high cellularity; and small cell change (tumor cells with scant cytoplasm relative to nuclear size) (62).

It has been documented that a Ki67 proliferation index over 4% has also been correlated with increased recurrence risk, however, it is most commonly used as an adjunct to standard WHO grading, rather than as an independent indicator of grade (64). As mentioned elevated mitoses and invasion are both regarded as sufficient for grade II classification, however, multiple grade II features can usually occur within the same atypical meningioma, i.e., invasion plus increased mitoses, sheet-like growth pattern and areas of high cellularity with small cell changes.

The other two subtypes of grade II meningiomas, clear cell and chordoid, may not show additional findings like elevated mitotic activity, necrosis and invasion and are in need of additional datasets to clarify their prognostic implications. Larger meningiomas require microscopic examination of several blocks to ensure lack of atypical features as well as absence of specialize variants (12). Assessment of brain invasion may also be apparent only by histologic evaluation, most often following thorough lower power scanning the periphery of the meningioma; an immunostain for glial fibrillary acidic protein can additionally be used to confirm minuscule foci of brain-invasion (12).

Grade III or anaplastic meningiomas can often resemble high-grade sarcomas, carcinomas or melanomas. While they often display *atypical* features of grade II lesions, the mitotic threshold differs, i.e., presence of > 20+ mitoses per 10 consecutive high-power fields. Thus, all meningiomas with 4–19 mitoses are still within the grade II spectrum. Rhabdoid and papillary morphologic variants are also considered to be grade III (12). Of meningioma with documented WHO grade, 80.5% were WHO grade I, 17.7% were WHO grade II, and 1.7% were WHO grade III (2).

### Genetics and Molecular Characteristics

The first genetic alteration found in association with meningiomas was observed by FISH in the deletion of Chromosome 22q, later determined to be the gene involved in NF2 on 22q12 (15, 65). The tumor suppressor, Merlin from 22q12 is inactivated in nearly two-thirds of meningiomas and is a member of the protein 4.1 superfamily of cytoskeleton linker proteins that includes erzin, radixin, and moesin (ERM) (14, 66, 67). Interestingly, *NF2* mutant meningiomas appear to have more histopathological findings of fibrous or transitional rather than some meningothelial histologic variants, likely due to lack of cytoskeleton linker resulting in a more mesenchymal phenotype (68, 69). Merlin is also involved in various developmental and survival signaling pathways with loss resulting in the dysregulation of cell proliferation, growth, and motility. Merlin enables Hippo-dependent YAP/TAZ destruction, restrains nuclear β-catenin activity in the WNT pathway, regulates TGF-β signaling activation, suppressor of mammalian target of the rapamycin (mTOR) pathway restricts activation of PGFR and EGFR, and controls the level of Notch receptor availability (70, 71). Therefore, it is not surprising that *NF2*-mutated meningiomas have been found to harbor more genetic alterations than the *NF2*-wildtype, despite both meningiomas within the same benign grade, which has continued the suggestion that a *NF2* mutation results in greater chromosomal instability overall (72).

Several studies have shown that a loss of 18q is associated higher WHO grade meningiomas and recurrence rates (73). The *DAL-1* (differentially expressed in adenocarcinoma of the lung) gene located at 18q has been purported to act as a potential tumor suppressor gene as a critical regulator of proliferation and apoptosis in meningiomas (74). Decreased expression of Dal-1 is also observed in up to 60%–76% of sporadic meningiomas, with loss of expression of either Dal-1 or merlin seen in 92% (75). The loss of merlin or Dal-1 are thought to be early events in the development or initiation of tumorigenesis in meningiomas (76). The loss of chromosome 10 has been found in a small study primarily in WHO Grade III but not in WHO Grade II specimens, suggesting that chromosome 10 loss may serve as a diagnostic and perhaps a prognostic marker (77).

Recent next-generation sequencing has elucidated a number of recurrent genetic alterations in *NF2*-nonmutated meningiomas which are driven by four mutually exclusive pathways: increased hedgehog signaling (through *SMO*, *SUFU* or *PRKAR1A* mutations); *TRAF7* (with either *KLF4* mutation or PI3K pathway activation); RNA polymerase II subunit A (*POLR2A*) mutations; and other (i.e., *AKT1)* mutations (68, 78). The majority of these mutations are usually found in WHO grade I meningiomas and also appear to not coexist with mutations in *NF2* (16). However, mutations in *TRAF7* can be present in isolation, though often they can co-occur with *KLF4*, *AKT1*, or *PIK3CA* mutations, whereas mutations in *SMO* and *POLR2A* are usually mutually exclusive (16, 79) Interestingly, the meningiomas arising from *SMO* and *AKT1-MTOR* aberrations often arise in the skull base (68). In contrast, meningiomas driven by the inactivation of *NF2* tend to localize primarily to the convexity (80). Likewise, there are associations between some mutations seen and with specific histopathologic variants of meningioma, for example *NF2* in fibroblastic and transitional meningiomas (68, 69), *KLF4* and *TRAF7* in secretory meningiomas (81), and *AKT1* mutations in grade I meningothelial meningiomas particularly of the base of the skull and spine (82). Mutations in BRAF V600E have been associated with rhabdoid meningiomas WHO grade III and recurrent meningiomas (83, 84). Alteration of the telomerase reverse transcriptase (*TERT*) promoter has been shown to be associated with an increased risk of recurrence (16, 85).

Growing evidence in the last two decades has shown that epigenetic modifications may have a pivotal function regarding tumorigenesis, progression and reoccurnce of meningiomas (**Table 4**) (86, 87). Moreover, several studies have propose methylation status of DNA within meningiomas may more accurately reflect the aggressiveness of the tumor and thus their anticipated recurrence rate compared with WHO grade of the lesion and/or extent of surgical excision (88–90). Numerous genes have been identified that are silenced by focal DNA hypermethylation in meningiomas include *TIMP3*, *TP73*, *MEG3*, *GSTP1*, several homeobox (*HOX*) family members (*HOXA7*, *HOXA9*, *HOXA10 HOXA6* and *HOXA9)*, *CDKN2A*, *WNK*, *TMEM30B*, and *MAL2* (91). In the case of hypermethylation of *TIMP3*, studies have shown that this methylation event inhibits matrix metalloproteinases and has been associated more aggressive and higher grade meningiomas (92, 93). Likewise, the inactivation of tumor suppressor gene, *TP73* by hypermethylation has been found in higher grade lesions and is thought to be associated with malignant transformation (94). Promoter methylation of *MEG3*, *GSTP1*, and *MAL2* has been shown to more commonly in higher grade meningiomas (92, 95, 96).

**Table 4:** Genes associated with meningiomas with corresponding chromosomal location and product (86).

Gene	Full name	Locus	Product
*NF2*	Neurofibromin 2	22q12.2	Merlin
*TRAF7*	TNF receptor-associated factor 7	16p13.3	TNF receptor-associated factor 7
*KLF4*	Kruppel-like factor 4	9p31	Kruppel-like factor 4
*AKT1*	v-Akt murine thymoma viral oncogene homolog 1	14q32.33	AKT1 kinase (serine/threonine protein kinase)
*SMO*	Smoothened, frizzled class receptor	7p32.1	Smoothened, G protein-coupled receptor
*PIK3CA*	Phosphadidylinositol-4,5-bisphosphate 3-kinase catalytic subunit alpha	3q26.32	Catalytic subunit of kinase, PI3K
*POLR2A*	RNA polymerase II subunit A	17p13.1	RNA polymerase II subunit A
*BAP1*	BRCA1-associated protein 1	3p21.1	Ubiquitin carboxyl-terminal hydrolase 1
*SMARCB1*	SWI/SNF related, matrix associated, actin dependent regulator of chromatin, subfamily b, member 1	22q11.23	Subunit of SWI/SNF complex
*SMARCE1*	SWI/SNF related, matrix associated, actin dependent regulator of chromatin, subfamily e, member 1	17q21.2	Subunit of SWI/SNF complex
*BRAF V600E*	B-Raf proto-oncogene	7q34	Serine/threonine kinase
*NOTCH2*	Notch receptor 2	1p12	Notch2 (notch receptor family)
*PTEN*	Phosphatase and tensin homolog	10q23.31	Phosphatidylinositol-3,4,5-triphosphate 3-phosphatase
*CDKN2A*	Cyclin-dependent kinase inhibitor 2A	9p21.3	p16(INK4A), p14(ARF)
*CDKN2B*	Cyclin-dependent kinase inhibitor 2B	9p21.3	p15(INK4B)

Various groups have subdivided meningiomas into distinct subsets based on the extent of the global DNA methylation profile, the have been various definitions but the results remained consistent which is the lesions within specific methylation classes (MCs) correlated particular mutations, histological variants, cytogenetic alterations and concluded that a DNA methylation-based classification system may provide a more accurate prognostication of clinical outcomes (88–90). For example, one group has been shown that WHO grade I meningiomas with intermediate level of methylation status have a worse clinical outcome than the average outcome of WHO grade I meningiomas (89). Similarly a WHO grade II meningiomas with a benign methylation classification profile appear to have an improved overall survival than the average of WHO grade II meningiomas (89). Taken together, one study has developed a DNA methylation-based model for predicting the risk of early (5-year) recurrence of meningiomas which combines the methylation status, with extent of resection and WHO grade in the hopes of tailoring ongoing surveillance and therapy (90).

Modifications in histones known to result in remodeling key complexes on chromatin have been reported for various malignancies in the recent years. It has been reported that meningiomas with the loss of trimethylation of lysine 27 of histone H3 (H3K27me3) *via* immunohistochemistry was associated with lesion that had documented rapid progression (97). In a large molecular profiling study have reported overexpression of the histone cluster H1 family member C (*HIST1HIc*) genes (6p) to be associated with recurrent meningiomas (98). In addition *HIST1Hic* has been shown mediate chromatin transcription by blocking chromatin acetylation (99) and aid maintenance or establishment of specific DNA methylation patterns (100). In addition, nearly 10% of non-*NF2* meningiomas harbor loss of function mutations of *KDM5C* and *KDM6A*, encoding histone lysine-specific demethylases, resulting in alterations in histone function and epigenetic regulation in meningiomas (68). As discussed earlier mutations of two core subunits of the SWI/SNF complex, *SMARCB1* and *SMARCE1*, have been identified in familial syndromes at risk of developing meningiomas (101). However, within anaplastic meningiomas the PRC2 histone methyltransferase complex, an antagonist of SWI/SNF complex, is upregulated result in aggressive disease and stemness and epithelial-to-mesenchymal transition (102).

There is increasing evidence for the role of microRNAs (miRNAs) as a regulator of epigenetic mechanisms as well as in the initiation, progression, and recurrence of meningiomas (103). For instance, some studies have shown that miR-200a may act as a tumor suppressor and that the downregulation of miR-200a may promote the development of meningiomas, as miR-200a has been found to be downregulated in meningiomas (104). In higher grade meningiomas, it has been shown that the downregulation of miRNA-145 has also indirectly associated with the overexpression of the *COL5A1* gene (encoding collagen type V alpha) thus miRNA-145 may account for the aggressive and invasive nature of these higher grade gliomas (105). Likewise, the upregulation of miR-21 has been demonstrated among WHO grade II or III meningiomas to a greater extent than that found in WHO grade I meningiomas (106). In meningiomas with high rates of recurrence it has been sown that there is an upregulation of miR-190a and downregulation of miR-29c-3p and miR-219-5p (107). The expression of miRNA-224 has been shown to correlate with advanced pathological grade and has been suggested that its expression could be used to predict the overall survival and recurrence-free survival of patients (106, 108).

### Treatment

#### Surgery

In an age of increased incidentally found meningiomas due to enhanced and improved imaging studies, when patients are asymptomatic, observation with routine surveillance imaging is an acceptable strategy. However, if the meningioma is growing and/or causing symptoms that could be related to the lesion, then maximal safe surgical resection is the standard of care. Nevertheless, the ability to achieve a GTR may be limited due to tumor location, involvement or invasion of nearby dural sinuses, arteries, cranial nerves and extent of brain invasion, especially in eloquent areas as well as patient specific factors affecting the safety of the procedure.

The surgical approach of meningiomas is dictated by the neuroanatomic location and surrounding structures. Convexity meningiomas are straightforward in their approach and often have GTRs. However, meningiomas in this location only account for about one sixth of meningiomas. Parasagittal meningiomas are more complex to resect and obtain a GTR as they often arise near the superficial sagittal sinus and can involve or invade this major intracranial draining sinus. In suspected cases of superficial sagittal sinus invasion, the surgical resection might not extend to remove that portion of the tumor due to an increased risk of air embolism, large blood loss and/or post-operative sinus thrombosis. Tumors of the skull base (sphenoid wing, olfactory groove, tuberculum sella, cerebellopontine angle or petroclival region) require more advanced surgical techniques and approaches to safely access the tumor without extensive brain retraction, injury to cranial nerve and vasculature. Advances in endoscopic technology and techniques have enabled the resection of skull bases meningiomas through an endoscopic endonasal approach that can be done alone or in combination with a traditional craniotomy, but risks associated with this location generally outweigh those in the convexity (109).

Several strategies might be leveraged pre- or intra-operatively for better outcomes. For example, coagulation and/or preoperative embolization could be employed to limit blood loss and to maintain good visualization throughout the procedure in hypervascular meningiomas. For meningiomas that are firm or calcified, a technique of debulking centrally or in piecemeal status through the resection can limit the need for retraction of the surrounding brain, cranial nerves, and corresponding vasculature. If the tumor forms a capsule in the arachnoid plane, performing the dissection while remaining in this plane can protect the pia of surrounding brain from injury. Similarly, cranial nerves and arteries may be enveloped or encased by skull base meningiomas, but the tumors rarely invade them and identification of the arachnoid plane can allow for safe dissection of the meningioma from normal structures. This technique of debulking, coagulation, and dissecting along the periphery are repeated until a GTR is achieved. As the adjacent dura is often involved with meningiomas, a dural graft is used in reconstruction. Additionally, the meningioma may invade adjacent bone of the skull. If involvement is limited, it may be possible to drill to the point of normal bone matrix; if there is more extensive involvement rendering the flap unsalvageable, the use of mesh or a cranial plating system instead should be considered. As mentioned, there are several factors that may preclude a GTR from occurring especially in skull base meningiomas (e.g., venous sinus involvement, arterial or cranial nerve envelopment and extensive involvement of the base of the skull). These circumstances may account at least in part for the improved survival of patients with convexity meningiomas over those with parasagittal and skull base meningiomas (110).

The extent of resection has been shown to be crucial to the rate of recurrence in the treatment of meningiomas. The extent of resection is defined by the Simpson grading system which is denoted by postoperative imaging as well as the assessment by the neurosurgeon during the procedure (**Table 5**) (111). A biopsy is a Simpson grade 5, subtotal resection of the meningioma is a grade 4, macroscopic resection without dural excision or coagulation is a Simpson grade 3, GTR with dural coagulation is a Simpson grade 2, and GTR including adjacent dura and bone is a Simpson grade 1 (111). Recurrence rates of Simpson grade I resection in a WHO grade I meningioma are low; they rise substantially with an increasing pathologic grade. In a retrospective study, 5 year recurrence rates after a Simpson grade I GTR in WHO grade I meningiomas are reported as 7%–23%, whereas the same resection in a WHO grade II results in a 50%–55% and in WHO grade III 72%–78% recurrence (9, 112). As the extent of resection decreases, there in an increase rate of recurrence (9, 113). However, the recurrence-free survival of Simpson grade 1–3 resection compared to Simpson grade 4 resection was more pronounced for tumors of the convexity than for parasagittal, parafalcine or skull base tumors as well as for meningiomas with high levels of proliferation (MIB-1 labeling index >3%) (114). Therefore, it is reasonable after a GTR of WHO grade I meningiomas to follow with routine surveillance imaging. However, in the case of a subtotal resection (Simpson grade 4–5) of WHO grade I meningioma, and generally all higher grade meningiomas comprised by WHO grade II and III, adjuvant treatment is necessary to delay or curtail recurrence.

**Table 5:** Simpson grade for surgical resection of meningiomas.

Simpson Grade	Definition (extent of resection)
Grade I	Complete removal including resection of underlying bone and associated dura
Grade II	Complete removal and coagulation of dural attachment
Grade III	Complete removal without resection of dura or coagulation
Grade IV	Subtotal resection
Grade V	Simple decompression with or without biopsy

#### Radiation Therapy

Radiation therapy has been the primary treatment for growing meningiomas that are deemed nonsurgically resectable based on location and/or patient co-morbidities which preclude surgical resection. Additionally, radiation therapy is employed as an adjuvant therapy after surgical resection, for recurrence after a resection, and some consider an upfront treatment approach if subtotal resection or operative morbidity is likely. Treatment can be delivered as a single-fraction stereotactic radiation (SRS) or fractionated external beam radiotherapy (EBRT). There is a scarcity of prospective studies comparing these different radiation therapy delivery regimens (techniques, doses, etc.) and comparing a single regimen to surgical resection, and therefore, most of the data is based on retrospective studies. Likewise, evaluating different radiation modalities *via* an outcome measure of recurrence rates or meningioma volume are plagued with over simplification of diverse meningioma population, genetics and treatment specific toxicities.

Treatment of recurrent WHO grade I and radiographically defined (presumed grade I) meningiomas is typically with a total dose of 50–54 Gy with a clinical target volume (CTV) margin of 0–5 mm (115, 116). For more advanced, WHO grade II-III meningiomas, treatment is typically 59.4–60 Gy with a wider 10–20 mm gross tumor volume (GTC) to CTV anisotropic expansion while respecting anatomic barriers to tumor growth (116). For smaller tumors with a diameter of less than 3–4 cm and at least 2-mm separation from critical normal structures (such as optic nerves), single fraction SRS is a feasible option. EBRT has been utilized for some tumor locations in which a GTR may cause significant morbidity to the patient (116). For example, one series of patients with optic nerve sheath meningiomas had ~25% of patients treated with EBRT alone and they showed no failures and improved or stable vision in 86% at a median of 8.3 years of follow-up (117). Another series of ~100 patients with presumed WHO grade I skull base meningiomas received EBRT only (65%) or following STR (35%), and showed local control of 95% for all patients at a median follow-up of 5 years (118). Studies of particle therapy are limited, although there are phase I and phase II trials underway to look at the role of proton radiation in a variety of settings with meningiomas [UPCC 24309 (NCT01117844)] (119). As well as a combined phase I/II study (NCT02693990) is investigating proton therapy with dose escalation for atypical meningiomas that underwent STR and anaplastic meningiomas following surgical resection (116). Brachytherapy is infrequently used, with the largest cohort being 42 patients receiving I-125 permanent seed implant during resection of atypical or malignant meningiomas. Eight-five percent of patients had a history of prior radiation therapy with a median time to progression of 11.4 months and numerous complications including radiation necrosis, wound breakdown, wound infection, and pseudomeningocele (116, 120). Ongoing studies will be needed to determine its utilization in the treatment of meningiomas.

The toxicities of radiation are dependent on the technique and dosing of radiation therapy implemented. EBRT toxicities are location dependent but are known to include alopecia. Side of effects of SRS are primarily limited to fatigue which is often transient and abated with a steroid regimen (119). Late toxicities for cranial radiation therapy include endocrinopathies, cognitive effects, increased cerebrovascular events, and secondary neoplasm risks as mentioned earlier (121). While the rate of these complications is low, they warrant discussion with patients given that the tumors are frequently benign.

When considering radiation therapy as a primary modality, there are several factors to take into account. First, radiation therapy is not as effective at relieving mass effect or tumor-associated edema, neurological deficits, or symptoms. However, if a patient is a poor surgical candidate or has lesions that are inaccessible for safe resection, radiation therapy is frequently employed for mitigation of local tumor growth. Second, the use of radiation therapy upfront precludes surgical biopsy, preventing histological confirmation of tumor grade and molecular features. This decreases the opportunity for targeted therapy, as well as limits understanding of the natural history of the meningioma and the risk of recurrence.

Add into the recent controversy of radiation therapy not as an adjuvant treatment but primary treatment, a recent RANO working group performed a systematic literature review; WHO Grade I meningiomas when treated to 50–54 Gy in 27–30 fractions EBRT had control rates of 87%–100%. Likewise, WHO grade I meningiomas treated with 12–16 Gy SRS had 10-year control rates greater than 90%, but this was location specific as parasellar and skull base meningiomas had lower rates of control (69%–90%) (9). The PFS in meningiomas less than 35 mm was better with SRS (mean dose 17.7 Gy) compared with Simpson grade 2–5 resection, although not for Simpson grade I (122). As expected, for larger meningioma volumes, there is decreased control especially in single fraction SRS as well as increased (5%–23%) radiation-related complications (123). Therefore, some centers hypofractionated SRS (up to 5 fractions) treatments for larger volume tumors, typically for those tumors >10 mm (3) which has abated some of the complications (edema and radiation necrosis) as well as mitigate development of toxicity by allowing repair of normal tissues (9, 124). Local control rates in which hypofractionated SRS has been utilized grade I and II meningioma was reportedly 95 and 71%, respectively, with no acute toxicities (125, 126). However, more studies need to conducted to determine the role of hypofractionated SRS in comparison to EBRT for similar pathological grades and sizes. Interestingly, a small study of patients that underwent either SRS or EBRT suggested that necrosis may be a negative predictor of radiation response regardless of radiation timing or modality (127).

For the majority of the cases, radiation therapy is adjuvant after surgical resection to decrease recurrence rates. In retrospective studies the addition of EBRT (to 59.4 Gy) demonstrated only 20% recurrence at 6 years versus 65% without radiation therapy following surgery (128). However, there is no consensus on the dosage and/or the timing of adjuvant radiation for high grade aggressive meningiomas. A recent cooperative group trial NRG/RTOG 0539 (NCT00895622) grouped patients into three risk categories in a nonrandomized fashion based on tumor grade and resection status. Patients with newly diagnosed grade I tumors following either gross total (Simpson grade 1–3) resection or subtotal (Simpson grade 4–5) resection were identified as being low-risk. This group showed a recurrence-free survival of 86% based on preliminary data. These findings support withholding adjuvant radiation for gross totally resected grade I tumors (129).

The National Comprehensive Cancer Network (NCCN) provides guidelines for the use of radiation therapy in the management of meningiomas, with most recommendations having Level 2A evidence (130). Radiation therapy should be considered for small (<30 mm) asymptomatic meningiomas at presentation if grade II and subtotally resected or grade III regardless of resection volume, and in grade I tumors when sub-totally resected if there is a ”potential” symptom. Radiation therapy should be pursued for large (>30 mm) asymptomatic tumors if grade III and considered if WHO grade II or incompletely resected grade I. For all asymptomatic meningiomas, observation alone (with serial imaging) is also an acceptable option. For symptomatic meningiomas at initial presentation, radiation therapy is recommended following surgery for any grade III and should be considered for any grade II tumors or large (>30 mm) incompletely resected grade I tumors. For surgically inaccessible tumors or surgically contraindicated patients, radiation treatment alone is also recommended. Upon recurrence, surgery (if accessible) followed by radiation treatment or re- radiation treatment, or radiation treatment alone (if inaccessible) is recommended (130). Of note, these guidelines do not take into account tumor location, patient age, or any molecular pathologic markers.

#### Systemic Treatment

As with radiation treatment there is a paucity of large and/or randomized trials to determine the efficaciousness of systemic therapy for the management of meningiomas. Thus, the NCCN recommends the use of only three classes of medical therapy: α-IFN, somatostatin receptor agonists and vascular endothelial growth factor (VEGF) inhibitors for the treatment of meningioma (130, 131). The guidelines by European Association of Neuro-Oncology (EANO) consider the use of systemic pharmacotherapy to be experimental with Level C evidence and thus do not recommend any specific agents or class of therapeutics for the management of meningiomas (**Table 6**) (132).

**Table 6:** Recommendations for the management of meningiomas of WHO grades I–III.

Histology, degree of resection	Recommendations for the therapeutic management
WHO grade I, gross total resection	Observation
WHO grade I, subtotal or partial resection	Observation or sterotactic radiosurgery/fractionated radiosurgery
WHO grade II, gross total resection	Observation or fractionated radiosurgery
WHO grade II, subtotal or partial resection	Fractionated radiosurgery
WHO grade III	Fractionated radiosurgery, experimental chemotherapy or peptide receptor radionuclide therapy

The utilization of IFN-α in the treatment of recurrent WHO grade I and in higher grade meningioma has shown some promise with PFS at 6 months of 54% and 17%, respectively (133, 134). However, these were small studies and IFN-α was moderately toxic, additional studies will need to performed to determine it efficacy.

However, there are more encouraging results with the use with antiangiogenic agents targeting VEGF. Sunitinib, a small molecule inhibitor of VEGF signaling was used in a Phase II trial of 36 patients with grade II/III refractory meningioma had a PFS at 6 months of 42%, however, had a high toxicity profile (60% with severe adverse events) (135). Bevacizumab, anti-VEGF monoclonal antibody, has been shown to have a PFS at 6 months of 87%, 77%, and 46% in recurrent grade I, II, and III tumors, respectively (136). A Phase II prospective trial of bevacizumab is ongoing (NCT01125046) for recurrent or progressive meningiomas.

Pasireotide, an alternative somatostatin analog, was utilized in a Phase II trial in recurrent meningioma that failed prior surgical or radiation treatment, although it only had a PFS at 6 months of 17% in the high grade (WHO grade II/III) cohort and 50% in the WHO grade I cohort and was well tolerated (137)). A recent retrospective chart review study, found that the use of sandostatin (octreotide) was especially effective in prolonging PFS at 6 month in estrogen negative progesterone positive tumors to 87.8% while patients with estrogen negative progesterone negative meningiomas had PFS at 6 months of 62.5% (138). However, in a trial of nine high grade meningioma patients treated with octreotide and in a larger trial of pasireotide, no radiographic response was observed and no significant benefit in PFS was detected (139).

Very much like gliomas, meningiomas often demonstrate immune evasion with T cell exhaustion resulting in decreased levels of PD-1^+^ T cells. However, trials of the inhibitory PD-L1 antibody-based therapies, prembrolizumab (NCT03016091, NCT03279692), nivolumab alone (NCT02648997), or nivolumab with hypofractionated SRS in combination with or without ipilumumab (CTLA4 inhibitor NCT03604978) and avelumab (in combination with proton radiotherapy, NCT03267836) are ongoing (12, 139). A recent case report demonstrated a remarkable response to nivolumab in a patient with recurrent, treatment-refractory meningioma and homozygous deletion of the DNA mismatch repair gene, MSH2 (140). Application of agents targeting the mTOR-pathway is currently being examined in trials with everolimus (NCT01880749 and NCT01419639) and vistusertib (AZD2014, NCT03071874, and NCT02831257). Everolimus is also being studied in combination with the somatostatin receptor analog octreotide (CAVOREM, NCT02333565) in recurrent meningioma (**Table 7**) (12, 139).

**Table 7:** Active recruiting of clinical trials for treatment of meningioma, updated and modified from Al-Rashed (139).

Drug (Trade Name)	Target	ClinicalTrials.gov Identifier
**Immunotherapies**		
Pembrolizumab (Keytruda)	PD-1	NCT03279692 NCT03016091
Avelumab and Hypofractionated Proton Radiation Therapy	PD-1	NCT03267836
Nivolumab (Opdivo) with or without Ipilimumab (Yervoy)	PD-1	NCT03604978
Nivolumab (Opdivo)	PD-1	NCT03173950
Targeted small molecules		
Vistusertib (AZD2014)	mTORC1/mTORC2	NCT03071874
Alpelisib (Piqray) and Trametinib (Mekinist)	PI3K/MEK	NCT03631953
Ribociclib	cyclin D1/CDK4 & CDK6	NCT02933736
Brigatinib (Alunbrig)	NF2	NCT04374305
Selumetinib	NF2	NCT03095248
Abemaciclib	CDK4/6	NCT03220646
		
Peptide receptor radionuclide therapy (PRRT)		
177Lu-DOTATATE (Lutathera)	SSR	NCT03971461 NCT04082520
		
Somatostatin receptor (SSTR)		
SOM230C	pasireotide LAR	NCT00859040
		
Tumor Treating Field		
NovoTTF-110A (Optune) and Bevacizumab (Avastin)	N/A	NCT02847559

## Conclusion

While meningiomas are a benign tumor, they nonetheless cause significant impact to patients and can challenge clinicians with their ongoing surveillance and management. Surgical resection remains the gold standard when GTR can be achieved. In cases where maximal resection cannot be obtained safely, inoperable cases, residual tumor remains, and/or the tumor is an aggressive high-grade lesion, adjuvant therapy is required. As reviewed, there are drawbacks to many of these adjuvant therapies and few systemic therapies have been approved or shown to be efficacious. Ongoing research and clinical trials will be needed to address these treatment gaps.

## Author Contributions

All authors listed have made a substantial, direct, and intellectual contribution to the work and approved it for publication.

## Conflict of Interest

The authors declare that the research was conducted in the absence of any commercial or financial relationships that could be construed as a potential conflict of interest.
